# Synergistic Suppression of Early Phase of Adipogenesis by Microsomal PGE Synthase-1 (PTGES1)-Produced PGE_2_ and Aldo-Keto Reductase 1B3-Produced PGF_2α_

**DOI:** 10.1371/journal.pone.0044698

**Published:** 2012-09-07

**Authors:** Ko Fujimori, Mutsumi Yano, Toshiyuki Ueno

**Affiliations:** Laboratory of Biodefense and Regulation, Osaka University of Pharmaceutical Sciences, Takatsuki, Osaka, Japan; University of Bari, Italy

## Abstract

We recently reported that aldo-keto reductase 1B3-produced prostaglandin (PG) F_2α_ suppressed the early phase of adipogenesis. PGE_2_ is also known to suppress adipogenesis. In this study, we found that microsomal PGE_2_ synthase (PGES)-1 (mPGES-1; PTGES1) acted as the PGES in adipocytes and that PGE_2_ and PGF_2α_ synergistically suppressed the early phase of adipogenesis. PGE_2_ production was detected in preadipocytes and transiently enhanced at 3 h after the initiation of adipogenesis of mouse adipocytic 3T3-L1 cells, followed by a quick decrease; and its production profile was similar to the expression of the cyclooxygenase-2 (PTGS2) gene. When 3T3-L1 cells were transfected with siRNAs for any one of the three major PTGESs, i.e., PTGES1, PTGES2 (mPGES-2), and PTGES3 (cytosolic PGES), only PTGES1 siRNA suppressed PGE_2_ production and enhanced the expression of adipogenic genes. AE1-329, a PTGER4 (EP4) receptor agonist, increased the expression of the *Ptgs2* gene with a peak at 1 h after the initiation of adipogenesis. PGE_2_-mediated enhancement of the PTGS2 expression was suppressed by the co-treatment with L-161982, a PTGER4 receptor antagonist. Moreover, AE1-329 enhanced the expression of the *Ptgs2* gene by binding of the cyclic AMP response element (CRE)-binding protein to the CRE of the *Ptgs2* promoter; and its binding was suppressed by co-treatment with L-161982, which was demonstrated by promoter luciferase and chromatin immunoprecipitation assays. Furthermore, when 3T3-L1 cells were caused to differentiate into adipocytes in medium containing both PGE_2_ and PGF_2α_, the expression of the adipogenic genes and the intracellular triglyceride level were decreased to a greater extent than in medium containing either of them, revealing that PGE_2_ and PGF_2α_ independently suppressed adipogenesis. These results indicate that PGE_2_ was synthesized by PTGES1 in adipocytes and synergistically suppressed the early phase of adipogenesis of 3T3-L1 cells in cooperation with PGF_2α_ through receptor-mediated activation of PTGS2 expression.

## Introduction

Obesity contributes to insulin resistance and type 2 diabetes mellitus [1], [2]. As a major target of insulin action, adipose tissue plays a critical role in the regulation of whole body metabolism and glucose homeostasis [3], [4]. Adipogenesis has been extensively studied, and several key transcription factors involved in the regulation of adipogenesis have been identified [5], [6]. Peroxisome proliferator-activated receptor (PPAR) γ plays a central role in this regulation [7], [8]. Ligand-activated PPARγ regulates many genes involved in glucose and lipid homeostasis and is involved in the maintenance of insulin responsiveness [8], [9], [10].

Prostaglandins (PGs) and their metabolites are involved in the regulation of adipogenesis. PGD_2_ [11] and its metabolite, Δ^12^-PGJ_2_ [12], activate the middle-late phase of adipogenesis, and PGD_2_-overproducing mice become obese under the high-fat diet [13]. Moreover, prostacyclin (PGI_2_) enhances adipogenesis through PGI_2_ receptor [14], [15]. In contrast, PGF_2α_ is produced by aldo-keto reductase (AKR) 1B3 in adipocytes; and it suppresses the early phase of adipogenesis through PTGFR receptors [16], [17]. PGF_2α_ promotes the production of anti-adipogenic PGF_2α_ and PGE_2_ by enhancing the expression of cyclooxygenase-2 (PTGS2; COX-2) through PTGFR (FP) receptor-activated mitogen-activated protein kinase/extracellular signal-regulated kinase kinase/extracellular signal-regulated kinase cascade and the binding of the cyclic AMP response element (CRE)-binding protein (CREB) to the CRE of the *Ptgs2* promoter [18]. Moreover, PGE_2_ is known to suppress adipogenesis by acting through the PTGER4 (EP4) receptor [19], and to increase the *de novo* synthesis of anti-adipogenic PGF_2α_ and PGE_2_ in mouse embryonic fibroblasts [20]. These anti-adipogenic PGs repress the function of PPARγ via their specific PG receptors.

Several PGE_2_ synthases (PTGESs) have been identified in various tissues [21], [22]. Microsomal PGES-1 (mPGES-1; PTGES1) is a member of the membrane-associated proteins in eicosanoid and glutathione metabolism (MAPEG) protein family [23], and produces PGE_2_ in response to various stimuli [24]. Microsomal PGES-2 (mPGES-2; PTGES2) has also been identified and its expression is high in the heart and brain [25]. Cytosolic PGES (cPGES; PTGES3) is constitutively and ubiquitously expressed in various cells [26]. However, the PGE_2_-producing enzyme in adipocytes has never been identified; and the mechanism causing suppression of the early-phase of adipogenesis by anti-adipogenic PGs such as PGE_2_ and PGF_2α_ remains unclear.

In this study, we demonstrate that PTGES1 was expressed in preadipocytes and that its mRNA and protein levels were consistently detected during adipogenesis. PGE_2_ production was detected in preadipocytes and increased during adipogenesis with a peak at 3 h after the initiation of adipogenesis, and PTGES1 was responsible for the production of PGE_2_ in adipocytes. PGE_2_ elevated the production of anti-adipogenic PGF_2α_ and PGE_2_ by enhancing the expression of PTGS2 by acting through the PTGER4 receptor, which action enhanced the binding of CREB to the *Ptgs2* promoter via activation of the PTGER4 receptor/CREB cascade in 3T3-L1 cells. Thus, PTGES1-produced PGE_2_ and AKR1B3-synthesized PGF_2α_ synergistically suppressed the early phase of adipogenesis through elevation of PTGS2 expression in 3T3-L1 cells.

## Materials and Methods

### Cell Culture

Mouse 3T3-L1 cells (Health Science Research Resources Bank, Osaka, Japan) were maintained in Dulbecco’s Modified Eagles Medium (DMEM; Sigma, St. Louis, MO, USA) supplemented with 10% (v/v) fetal calf serum and antibiotics. The cells were maintained in a humidified atmosphere of 5% CO_2_ at 37°C.

Adipocyte differentiation of 3T3-L1 cells was initiated by incubation for 2 days in DMEM containing insulin (10 µg/ml; Sigma), 1 µM dexamethasone (Sigma), and 0.5 mM 3-isobutyl-1-methylxanthine (Sigma). On day 2, the medium was replaced with DMEM containing insulin (10 µg/ml) alone and changed every 2 days.

Oil Red O staining was carried out as described previously [11]. Spectrophotometric measurement for Oil Red O staining was performed by dissolving the stained lipid droplets in the cells with isopropyl alcohol, and then the absorbance was measured at 520 nm.

### RNA Preparation and Quantification of RNA

Total RNA was extracted with Sepasol-RNAI (Nacalai Tesque, Kyoto, Japan), followed by further purification with an RNeasy Purification System (Qiagen, Hilden, Germany) [17]. The first-strand cDNAs were synthesized from 1 µg of total RNA with random hexamer and ReverTra Ace Reverse Transcriptase (Toyobo, Osaka, Japan) at 42°C for 60 min after initial denaturation at 72°C for 3 min, followed by heat-denaturation of the enzyme at 99°C for 5 min. The cDNAs were further utilized as the templates for quantitative PCR analyses.

Expression levels were quantified by using a LightCycler system (Roche Diagnostics, Mannheim, Germany) with THUNDERBIRD qPCR Mix (Toyobo) and primer sets (Table 1). The expression level of the target genes was normalized to that of the TATA-binding protein (TBP).

**Table 1:** Nucleotide sequence of primers used in this study.

Gene	Acc No.	Forward primer	Reverse primer
Pparγ	NM_001127330	5′-CAAGAATACCAAAGTGCGATCAA-3′	5′-GAGCTGGGTCTTTTCAGAATAATAAG-3′
C/ebpα	NM_007678	5′-CTGGAAAGAAGGCCACCTC-3′	5′-AAGAGAAGGAAGCGGTCCA-3′
Fabp4	NM_024406	5′- CAGCCTTTCTCACCTGGAAG-3′	5′- TTGTGGCAAAGCCCACTC -3′
Scd1	NM_009127	5′- TTCCCTCCTGCAAGCTCTAC-3′	5′- CAGAGCGCTGGTCATGTAGT-3′
Ptges1	NM_022415	5′-GCACACTGCTGGTCATCAAG-3′	5′-ACGTTTCAGCGCATCCTC-3′
Ptges2	NM_133783	5′-CCCAGGAAGGAGACAGCTT-3′	5′-AGGTAGGTCTTGAGGGCACTAAT-3′
Ptges3	NM_019766	5′-CGAATTTTGACCGTTTCTCTG-3′	5′-TGAATCATCATCTGCTCCATCT-3′
Ptgs2	NM_011198	5′-GATGCTCTTCCGAGCTGTG-3′	5′-GGATTGGAACAGCAAGGATTT-3′
Ptger1	NM_013641	5′-GAGCCAGGGAGTAGCTGGA-3′	5′-GCTCATATCAGTGGCCAAGAG-3′
Ptger4	NM_001136079	5′- CCTAACCCCACCCTACAGGT-3′	5′-AGAAGGACGCGTTGACTCC-3′
Tbp	NM_013684	5′-GTGATGTGAAGTTCCCCATAAGG-3′	5′-CTACTGAACTGCTGGTGGGTCA-3′

### Suppression by RNAi

PTGES1 Stealth siRNA (5′-UUCUUCCGCAGCCUCAUCUGGCCUG-3′) and Stealth Negative Control (N.C.) siRNA were obtained from Invitrogen (Carlsbad, CA, USA). SiRNAs for PTGES2 (5′-CUGUACUUGCCUCCUCUAACC-3), PTGES3 (5′-GGUAGACCUCUAUGAAGCAGC-3′) and MISSION siRNA Universal Negative Control were purchased from Sigma Genosys (Sapporo, Japan). Transfection with siRNA (20 nM) was performed by use of X-tremeGENE siRNA Transfection Reagent (Roche Diagnostics, Mannheim, Germany) according to the manufacturer’s instructions. Transfection efficiency of siRNA was measured by using FAM-labeled GAPDH siRNA (Ambion, Austin, TX, USA), and we found it approximately 50–60% in 3T3-L1 cells (data not shown).

For determination of the knockdown-efficiency of each siRNA, 3T3-L1 cells were transfected with each siRNA and cultured for 2 days.

For identification of the functions of PTGESs, 3T3-L1 cells were transfected with each siRNA, and caused to differentiate into adipocytes for 6 days. Transfection with siRNA was carried out every 2 days.

### Western Blot Analysis

Cells were harvested and lysed in RIPA buffer containing 50 mM Tris-Cl, pH 8.0, 150 mM NaCl, 0.1%(w/v) SDS, 0.5%(w/v) sodium deoxycholate, 1%(v/v) NP-40, and 1%(v/v) Triton X-100 with Protease Inhibitor cocktail (Nacalai Tesque) and phosphatase inhibitors; 50 µM Na_2_MoO_4_, 1 mM NaF and 1 mM Na_3_VO_4_. The lysates were centrifuged for 20 min at 12,000×*g* at 4°C to remove the cell debris. Protein concentrations were measured with a Pierce BCA Protein Assay Reagent (Thermo Scientific, Rockford, IL, USA). The proteins were separated on SDS-PAGE gels and transferred onto PVDF membranes (Immobilon P; Millipore, Bedford, MA, USA). The blots were incubated with a given antibody: anti-PTGES1 (1∶1,000; Cayman Chemicals, Ann Arbor, MI, USA), anti-PTGES2 (1∶1,000; Cayman Chemicals), anti-PTGES3 (1∶1,000; Cayman Chemicals), anti-PTGS2 (C-20; 1∶500; Santa Cruz Biotech., Santa Cruz, CA, USA), anti-PPARγ (H-100; 1∶1,000; Santa Cruz Biotech.) or anti-stearoyl-CoA desaturase (SCD1; S-15; 1∶1,000; Santa Cruz Biotech.) polyclonal antibody, or anti-fatty acid binding protein 4 (FABP4; EPR3579; 1∶1,000; Epitomics, Burlingame, CA, USA) or anti-actin (AC-15; 1∶2,000; Sigma) monoclonal antibody. After the blots had been washed with TBS-T {20 mM Tris, 137 mM NaCl, 0.1%(v/v) Tween-20, pH 7.6}, they were incubated with anti-rabbit, anti-goat or anti-mouse IgG antibody conjugated with horseradish peroxidase (Santa Cruz Biotech.). Immunoreactive signals were detected with an Immobilon Western Detection Reagent (Millipore) by use of an LAS-3000 Image analyzer (Fujifilm, Tokyo, Japan) and analyzed with MultiGauge software (Fujifilm). Each expression level was normalized by that of actin.

### Measurement of PGs

PGE_2_ and PGF_2α_ levels were measured by use of their respective enzyme immunoassay kit (EIA; Cayman Chemical) as described previously [17]. In brief, cells were treated with A23187 (5 µM; Calbiochem, San Diego, CA, USA), a calcium ionophore, for 10 min at 37°C. Medium was collected, and centrifuged at 3,000×*g* for 5 min to remove the cells. The resultant supernatant was then used for measurement of PGE_2_ and PGF_2α_ by performing their respective EIA according to the manufacturer’s instructions.

### Measurement of Triglyceride Level

Cells were washed with PBS, and lysed with PBS containing 5%(v/v) Triton-X100, and then incubated at 90°C for 3 min. The supernatant was prepared by centrifugation to remove cell debris, and subsequently used for measurement of the intracellular triglyceride level by using a WAKO LabAssay Triglyceride Kit (Wako Pure Chemical, Osaka Japan) according to the manufacturer’s instructions. Absorbance was measured at 570 nm. Protein concentrations were measured as described above.

### Luciferase Reporter Assay

The luciferase reporter vectors carrying the mouse *Ptgs2* promoter were generated previously [18]. 3T3-L1 cells were co-transfected with each construct (0.9 µg) and pRL-SV40 (0.1 µg, Promega, Madison, WI, USA) in 6-well plates, the latter plasmid carrying the *Renilla* luciferase gene under the control of the SV40 promoter as the transfection control, along with FuGENE6 Transfection Reagent (Roche Diagnostics) according to the manufacturer’s instructions. The cells were cultured for a further 48 h in the presence or absence of AE1-329 (1 µM) or L-161982 (10 µM), and the luciferase activities were measured by using a Dual-Glo Luciferase Assay System (Promega). The reporter activity was calculated relative to that of pGL4.10[luc2] vector (Promega), which was defined as “1”. Data were obtained from three independent experiments, and each experiment was performed in triplicate. The relative promoter activities were presented as the mean ± S.D.

### Chromatin Immunoprecipitation (ChIP) Assay

The ChIP assay was performed as described previously [11] by using anti-CREB polyclonal antibody (H-74; Santa Cruz Biotech.). Immunoprecipitated DNA-protein complexes were reverse-crosslinked, and the free DNAs were purified by ethanol-precipitation and utilized for subsequent PCR amplification with KOD FX DNA Polymerase (Toyobo) with a primer set specific for CRE at position -59 in the *Ptgs2* promoter: 5′-CAGAGAGGGGGAAAAGTTGG-3′ and 5′-GAGCAGAGTCCTGACTGACTC-3′. PCR was conducted under the following conditions: initial denaturation at 94°C for 2 min, followed by 30 cycles of 98°C for 10 sec, 55°C for 20 sec, and 60°C for 20 sec. The amplified PCR products (expected size of 168-bp) were analyzed by performing agarose gel electrophoresis, followed by staining of the gels with ethidium bromide.

In addition, we performed the quantitative PCR analysis to measure the anti-CREB antibody-precipitated DNA level by using the same primers used in PCR analysis as described above. Briefly, the precipitated DNA level was estimated by the use of serially diluted concentration-known DNA including the *Ptgs2* promoter region as the standard.

### Statistical Analysis

Comparison of 2 groups was analyzed by Student’s *t*-test. For comparison of more than 2 groups with comparable variances, one-way ANOVA and Tukey’s *post-hoc* test was carried out. *p*<0.05 was considered significant.

## Results

### Identification of PGES in Adipocytes

**Figure 1:**
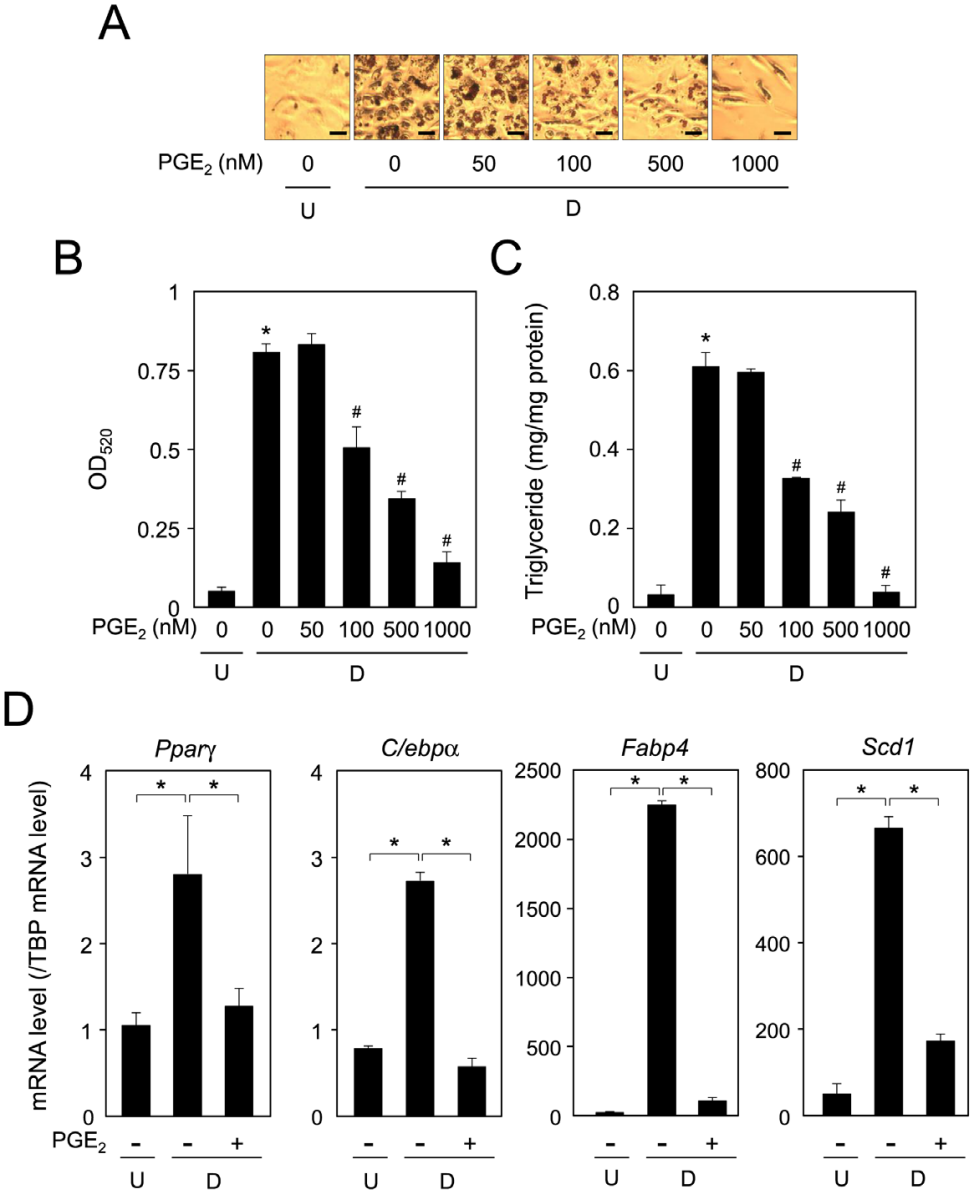
Suppression of adipogenesis by PGE_2_ in 3T3-L1 cells. A. Oil Red O staining of PGE_2_-treated 3T3-L1 cells. Cells (undifferentiated cells: U) were caused to differentiate into adipocytes (D) for 6 days in medium containing various concentrations of PGE_2_ (0–1000 nM; Cayman Chemical). Intracellular lipid droplets were stained with Oil Red O. Bar  = 50 µm. B. Measurement of Oil Red O dye extracted from lipid droplet-laden cells. *p<0.01 as compared to undifferentiated cells. ^#^ *p*<0.01 as compared to vehicle-treated differentiated cells. C. PGE_2_-suppressed intracellular triglyceride level of 3T3-L1 cells. Cells were cultured as described in the legend of Fig. 1A. Data are presented as the mean ± S.D. from 3 independent experiments. *p<0.01 as compared to undifferentiated cells. ^#^ *p*<0.01 as compared to vehicle-treated differentiated cells. D. Expression level of adipogenic genes in PGE_2_-treated and non-treated 3T3-L1 cells. Cells were caused to differentiate into adipocytes in medium with or without PGE_2_ (100 nM). Messenger RNA levels were measured by quantitative PCR. The data are presented as the mean ± S.D. from 3 independent experiments. *p<0.01 as indicated by the brackets.

At first, we investigated the suppression of adipogenesis by treatment with PGE_2_. Mouse 3T3-L1 cells were caused to differentiate into adipocytes for 6 days in the presence of various concentrations of PGE_2_. Oil Red O staining showed that intracellular lipid-droplets increased in size and number during adipogenesis, and these enhancements were repressed in a PGE_2_-concentration-dependent manner (Fig. 1A and 1B). Moreover, the intracellular triglyceride level was also enhanced during adipogenesis, and when the cells were caused to differentiate into adipocytes in the presence of various concentrations of PGE_2_, its level was significantly repressed in a concentration-dependent manner (Fig. 1C). When the cells were cultured in medium containing various concentrations of PGE_2_ for 3 h, followed culture for 6 days in the absence of PGE_2_, suppression effect at 3 h was slightly weaker than those for 6 days (data not shown). Furthermore, the expression level of adipogenic genes such as total *Pparγ* (*Pparγ1* and *Pparγ2*), CCAAT-enhancer binding protein (*C/ebp*)α, *Fabp4*, and *Scd1* in adipocytes was elevated approximately 2.8-, 3.6-, 59.2-, and 8.9-fold, respectively, as compared with each of those of the undifferentiated cells (Fig. 1D). Furthermore, the enhanced expression of these genes was suppressed by about 57, 74, 96, and 88%, respectively, of that of the vehicle-treated differentiated cells (Fig. 1D). These results indicate that PGE_2_ suppressed adipogenesis measured in terms of the expression of adipogenic genes in 3T3-L1 cells.

**Figure 2:**
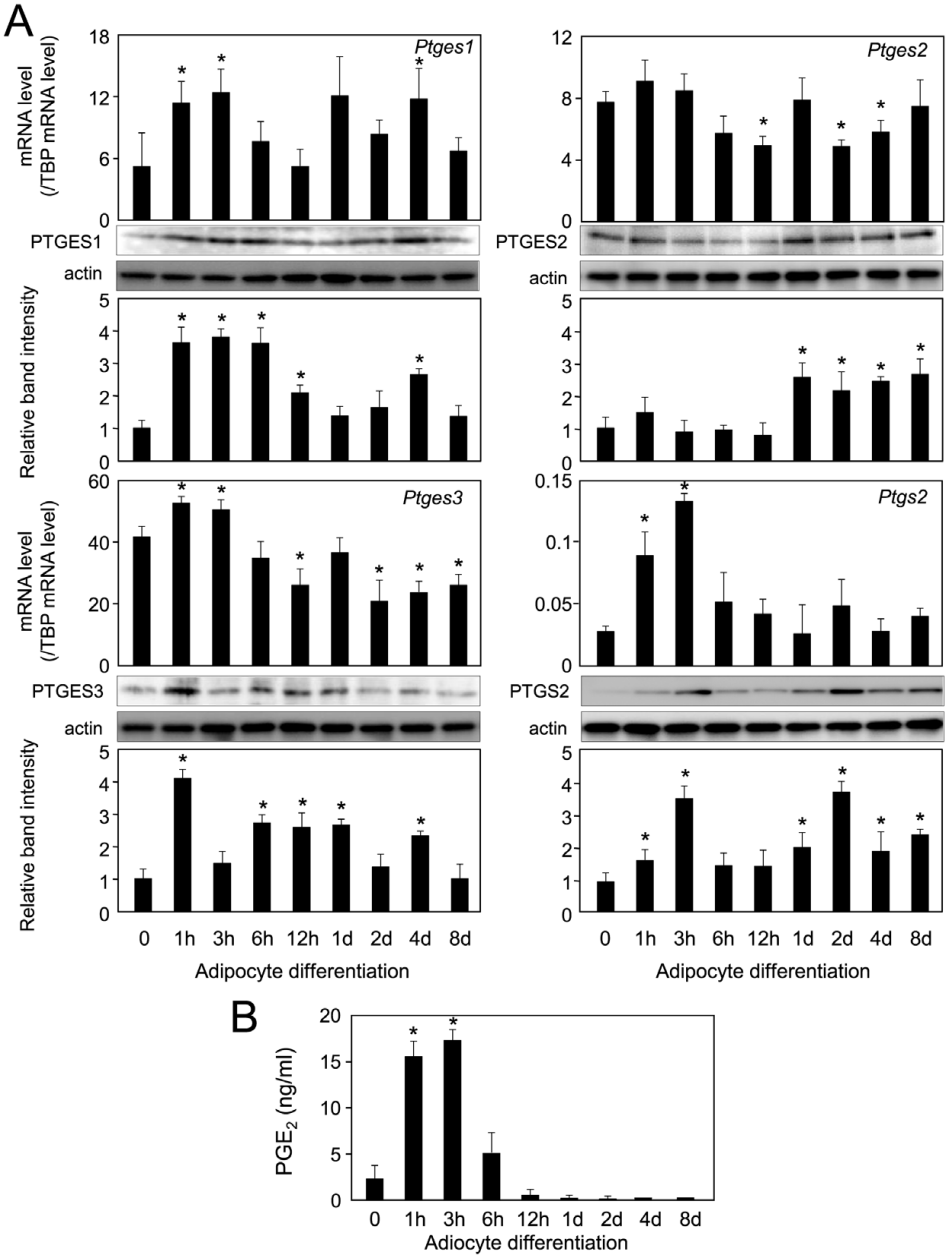
Expression of PTGESs and PTGS2 in 3T3-L1 cells. A. Expression of three PTGESs and PTGS2 during the differentiation of 3T3-L1 cells. The expression level of each gene was measured by quantitative PCR. The data are presented as the mean ± S.D. from 3 independent experiments. *p<0.01 as compared with value for undifferentiated cells. Protein levels were detected by Western blot analysis by use of crude cell extracts (20 µg/lane). Band intensities were measured by using MultiGauge software, and normalized by actin level. Data are representative of 3 independent experiments and presented as the mean ± S.D. *p<0.01, as compared to undifferentiated cells. B. The PGE_2_ level was measured by EIA. *p<0.01 as compared to undifferentiated cells.

**Figure 3:**
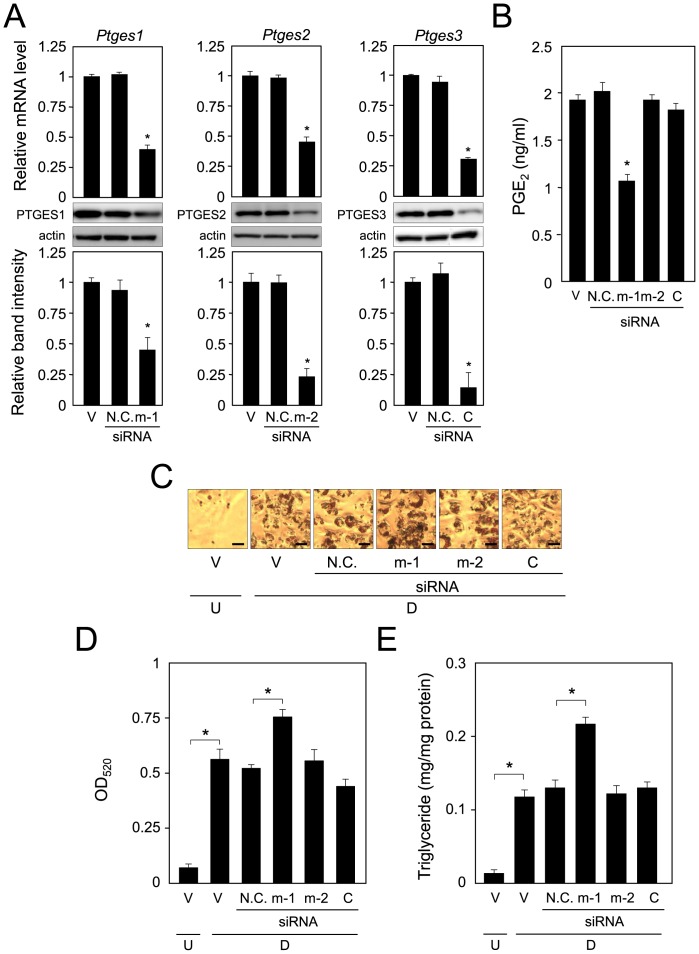
Adipogenesis in *Ptges*-knockdown cells. A, siRNA-mediated suppression of the expression of the *Ptges* genes. 3T3-L1 cells (V; vehicle) were transfected with an siRNA for the *Ptges1* (m-1), *Ptges2* (m-2) or *Ptges3* (C) gene or with N.C. siRNA (N.C.), and caused to differentiate into adipocytes for 2 days. Each gene expression was measured by quantitative PCR. Data are presented as the mean ± S.D. from 3 independent experiments. *p<0.01 as compared with value for N.C. siRNA. Protein levels were detected by Western blot analysis using crude cell extracts (20 µg/lane). Band intensities were measured by using MultiGauge software, and normalized by actin level. Data are representative of 3 independent experiments and presented as the mean ± S.D. *p<0.01, as compared with N.C. siRNA-transfected cells. B. PGE_2_ production in *Ptges*-knockdown cells. 3T3-L1 cells were transfected with each siRNA described in the legend of Fig. 3A, and the PGE_2_ level was measured by EIA. *p<0.01, as compared with value for N.C. siRNA. C. Oil Red O staining of *Ptges*-knockdown cells. 3T3-L1 cells (undifferentiated cells: U) were transfected with siRNA and caused to differentiate into adipocytes for 6 days (D). Transfection was carried out every 2 days. Bar  = 50 µm. D. Measurement of Oil Red O dye extracted from lipid droplet-laden cells. E. Intracellular triglyceride level in *Ptges*-knockdown cells. Cells were cultured as described in the legend of Fig. 3C. Data are presented as the mean ± S.D. from 3 independent experiments. *p<0.01, as indicated by the brackets.

Next, we examined the expression of the *Ptges* genes in 3T3-L1 cells. Cells were caused to differentiate into adipocytes for 6 days, and the gene expression of the three major PTGESs, i.e., PTGES1, PTGES2, and PTGES3, during adipogenesis was measured by performing quantitative PCR and Western blot analyses. All three PTGESs were expressed in preadipocytes and consistently so during adipogenesis (Fig. 2A). The protein levels of all three PTGESs, examined by Western blot analysis, well resembled those of their mRNA expression (Fig. 2A). However, the expression profiles of the *Ptgs2* gene and protein were quite different from them; as it was transiently up-regulated at 3 h after the initiation of adipogenesis, and then decreased to the basal level (Fig. 2A). Then, we measured the PGE_2_ level during adipogenesis by EIA. PGE_2_ was produced in preadipocytes, and its production level rapidly increased to a peak at 3 h after initiation of adipogenesis and then quickly decreased to a level lower than that of the undifferentiated cells (Fig. 2B). This production pattern well resembled the expression profile in the PTGS2 (Fig. 2A). These results reveal that all three PTGESs were consistently expressed during adipogenesis. PGE_2_ production was detected in preadipocytes and transiently enhanced at 3 h after the initiation of adipogenesis, whose pattern well resembled the expression of PTGS2 in 3T3-L1 cells.

**Figure 4:**
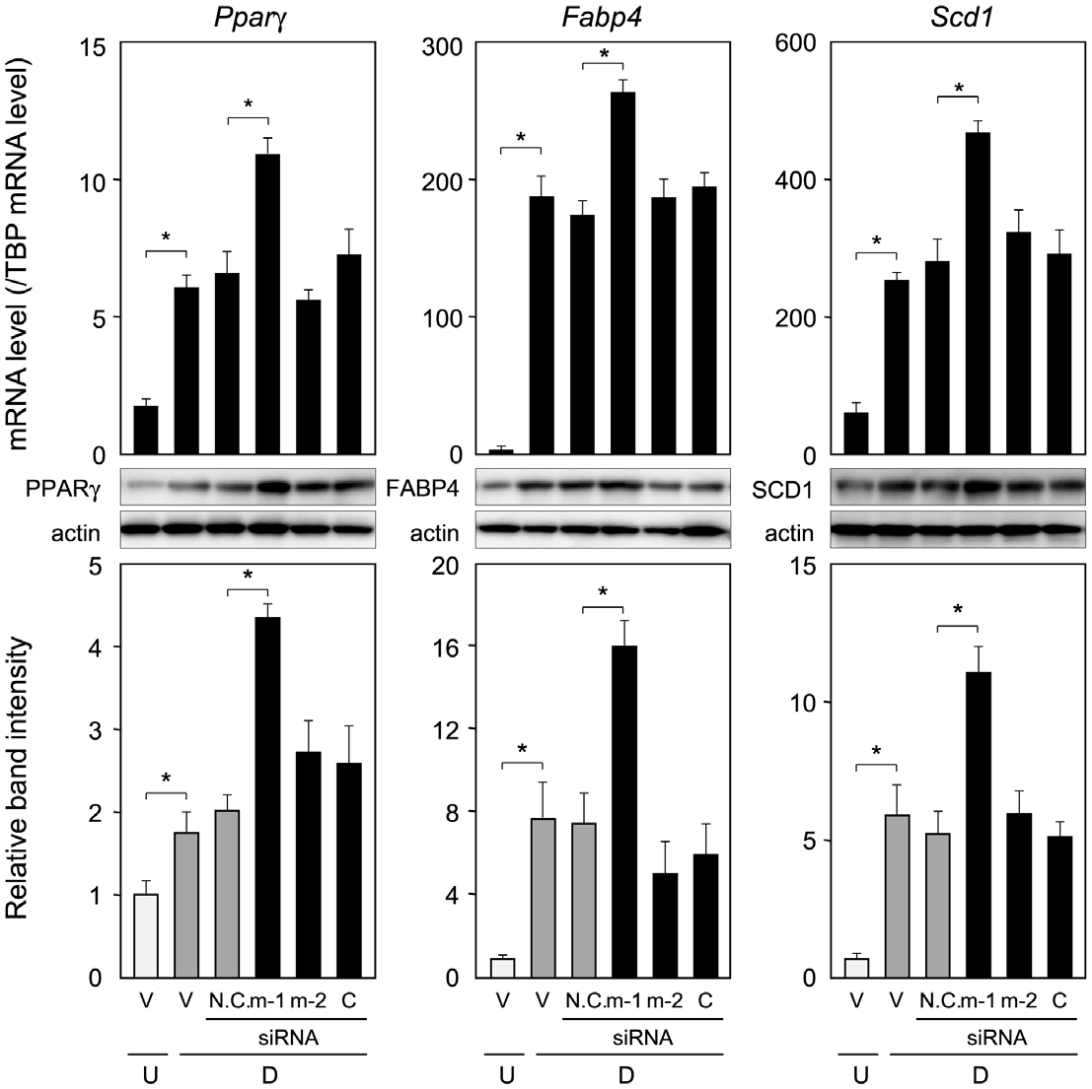
Expression of adipogenic genes in *Ptges*-knockdown cells. Cells were cultured as described in the legend of Fig. 3C, and mRNA levels were measured by quantitative PCR. The data are presented as the mean ± S.D. from 3 independent experiments. *p<0.01, as indicted by the brackets. Protein levels were detected by Western blot analysis using crude cell extracts (20 µg/lane). The data are the representative of 3 independent experiments. *p<0.01, as indicated by the brackets.

### PTGES1 is Responsible for the Production of PGE_2_ in Adipocytes

To identify the active PGES in adipocytes, we transfected 3T3-L1 cells separately with each of the PTGES siRNAs, and differentiated into adipocytes for 2 days. The mRNA levels of all three *Ptges* genes were significantly decreased more than 50% by their respective siRNAs, as compared with each of their levels when treated with N.C. siRNA (Fig. 3A). Almost the same results were obtained at the protein level by Western blot analysis; and the actin level, as the internal control, was almost the same in all samples (Fig. 3A). Each siRNA was specific for its PTGES, as it did not inhibit the expression of the other *Ptges* mRNAs (data not shown). Moreover, the siRNA for PTGES1 decreased the PGE_2_ production to about 61.4% of that with N.C. siRNA in 3T3-L1 cells (Fig. 3B). In contrast, siRNAs for PTGES2 and PTGES3 did not have any effect on the production of PGE_2_ (Fig. 3B); although the mRNA and protein levels of PTGES2 and PTGES3 were significantly decreased by their respective siRNAs (Fig. 3A).

**Figure 5:**
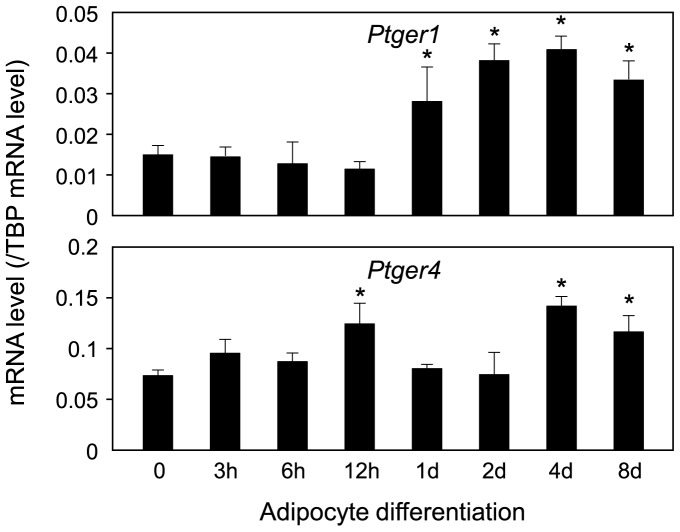
Expression level of *Ptger1* and *Ptger4* receptor genes during adipogenesis of 3T3-1 cells. Expression levels of the *Ptger1* and *Ptger4* genes during adipocyte differentiation of 3T3-L1 cells. Cells were caused to differentiate into adipocytes for 8 days. Transcription levels were measured by quantitative PCR. The data are presented as the mean ± S.D. from 3 independent experiments. *p<0.01, as compared with value for undifferentiated cells (0 hr).

To confirm that PTGES1 is the PGES suppressing adipocyte differentiation, we examined the role of PGES in the accumulation of intracellular lipids. As shown above, PGE_2_ inhibited the accumulation of intracellular lipids of 3T3-L1 cells (Fig. 1A and 1B). When the cells were transfected with any one of the three PTGES siRNAs, the intracellular lipid level demonstrated by Oil Red O staining was increased only in the PTGES1 siRNA-transfected cells (Fig. 3C and 3D). Whereas, there were no changes in PTGES2 or PTGES3 siRNA-transfected cells, which showed almost the same lipid accumulation as the vehicle-treated cells (Fig. 3C and 3D). In addition, the intracellular triglyceride level in PTGES1 siRNA-transfected cells was clearly increased as compared with that in the vehicle-treated or PTGES2 or PTGES3 siRNA-transfected cells (Fig. 3E), indicating that PTGES1 was involved in the accumulation of the intracellular triglyceride level in adipocytes.

**Figure 6:**
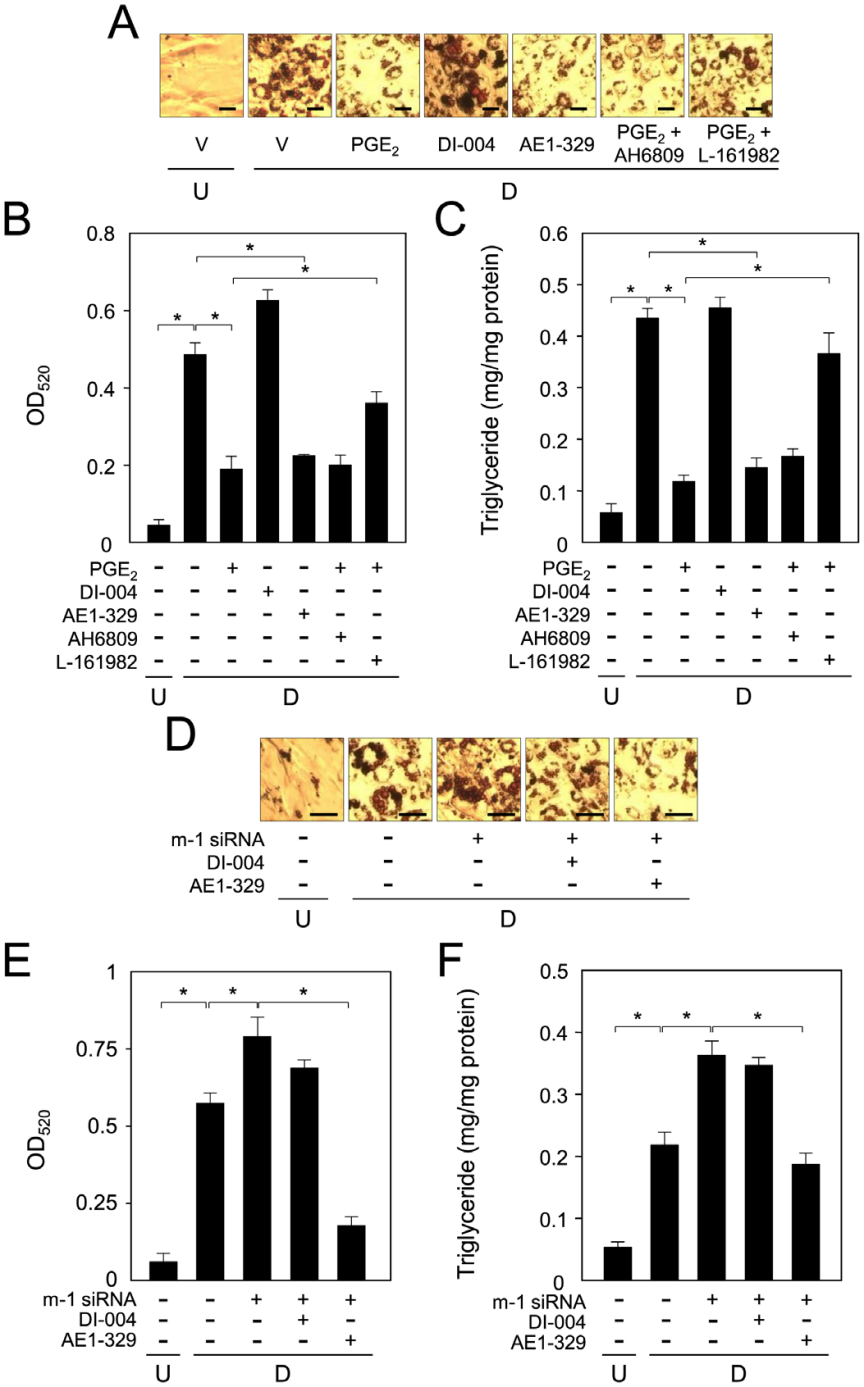
PGE_2_ suppresses adipogenesis through PTGER4 receptors. A. Accumulation of lipid-droplets through PTGER4 receptor action. 3T3-L1 cells (undifferentiated cells: U) were caused to differentiate into adipocytes (D) for 6 days in medium containing either PGE_2_ (100 nM), DI-004 (PTGER1 receptor agonist; 1 µM; ONO Pharmaceutical) or AE1-329 (PTGER4 receptor agonist; 1 µM; ONO Pharmaceutical) or PGE_2_ and AH6809 (PTGER1 receptor antagonist; 1 µM; Cayman Chemical) or L-161982 (PTGER4 receptor antagonist; 10 µM; Cayman Chemical). Bar  = 50 µm. B. Measurement of Oil Red O dye extracted from lipid droplet-laden cells. C. Effects of PGE_2_ and PTGER agonists/antagonists on the intracellular triglyceride level. 3T3-L1 cells were cultured as described in the legend of Fig. 7A. Data are presented as the mean ± S.D. from 3 independent experiments. *p<0.01, as indicated by the brackets. D. Accumulation of lipid-droplet by PTGES1-produced PGE_2_ acting via the PTGER4 receptor. 3T3-L1 cells (undifferentiated cells: U) were transfected with PTGES1 siRNA (m-1 siRNA) and caused to differentiate into adipocytes (D) for 6 days in medium containing either DI-004 (1 µM) or AE1-329 (1 µM). Bar  = 50 µm. E. Measurement of Oil Red O dye extracted from lipid droplet-laden cells. F. Effects of PTGES1 siRNA and PTGER agonists on intracellular triglyceride level. 3T3-L1 cells were cultured as described in the legend of Fig. 6D. Data are presented as the mean ± S.D. from 3 independent experiments. *p<0.01, as indicated by the brackets.

Next, we investigated the expression of adipogenic genes in PTGES siRNA-transfected cells. The transcription level of the adipogenic genes such as *Pparγ*, *Fabp4*, and *Scd1* was enhanced approximately 1.7-, 1.4-, and 1.6-fold, respectively, by transfection with PTGES1 siRNA, as compared with those levels for cells treated with vehicle or transfected with N.C. siRNA, PTGES2 siRNA or PTGES3 siRNA (Fig. 4). Protein levels of PPARγ, FABP4, and SCD1 were also up-regulated by transfection of 3T3-L1 cells with PTGES1 siRNA, but not affected in N.C., PTGES2 or PTGES3 siRNA-transfected cells (Fig. 4). These results reveal that PTGES1 acted as the PGES in adipocytes and that PTGES1-produced PGE_2_ suppresses adipogenesis by reducing the expression of adipogenic genes in 3T3-L1 cells.

**Figure 7:**
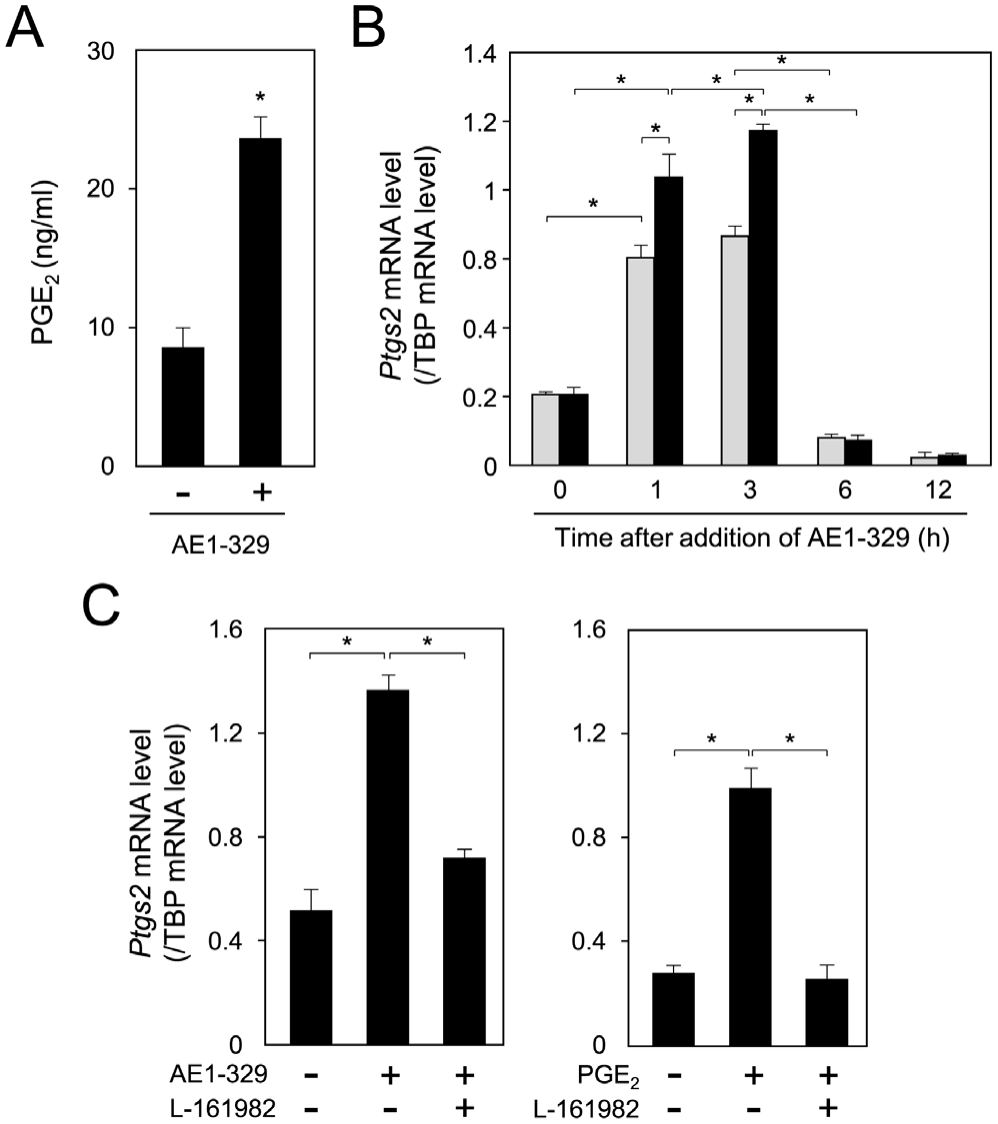
Enhancement of *Ptgs2* expression by activation of PTGER4 receptor in 3T3-L1 cells. A. Elevation of PGE_2_ production by treatment with AE1-329 in 3T3-L1 cells. Cells were incubated with AE1-329 (1 µM) or vehicle for 1 h, followed by treatment with A23187 (5 µM) for 10 min at 37°C, after which the medium was collected to measure the PGE_2_ level by EIA. *p<0.01, compared with value for vehicle-treated cells. B. Change in the transcription level of the *Ptgs2* gene in the presence (*black columns*) or absence (*gray columns*) of AE1-329. 3T3-L1 cells were incubated with or without AE1-329 (1 µM) for various times. The expression level of the *Ptgs2* gene was measured by quantitative PCR. The data are presented as the mean ± S.D. of 3 independent experiments. *p<0.01, compared with the value for the untreated cells (0 h). C. AE1-329- or PGE_2_-mediated upregulation of *Ptgs2* gene expression through PTGER4 receptor. 3T3-L1 cells were caused to differentiate into adipocytes for 6 days in medium containing AE1-329 (1 µM) or PGE_2_ (100 nM) with or without L-161982 (10 µM). *Ptgs2* mRNA levels were measured by quantitative PCR. The data are presented as the mean ± S.D. of 3 independent experiments. *p<0.01, as indicated by the brackets.

### Involvement of PTGER4 Receptors in the Suppression of Adipogenesis

PGE_2_ exerts its action through interaction with four PGE_2_ receptor subtypes; PTGER1 (EP1), PTGER2 (EP2), PTGER3 (EP3), and PTGER4 (EP4) [27]. So next, we investigated the expression of the *Ptger* genes during adipocyte differentiation of 3T3-L1 cells. *Ptger1* mRNA was detected in preadipocytes and its expression level gradually increased after 1 day of the initiation of the adipocyte differentiation (Fig. 5). The *Ptger4* gene was expressed highly in preadipocytes, and its level decreased almost 50% after the initiation of adipogenesis (Fig. 5). Whereas, the expression of *Ptger2* and *Ptger3* receptor genes was under the detection limit of our experimental conditions (data not shown).

Next, we examined which PTGER receptor, PTGER1 or PTGER4 was involved in the PGE_2_-mediated suppression of adipogenesis. When 3T3-L1 cells were caused to differentiate into adipocytes for 6 days in medium containing a PTGER1 receptor agonist, DI-004, the accumulation of lipid-droplet in the cells was not changed as judged by Oil Red O staining (Fig. 6A and 6B). Whereas, the amount of lipid-droplets was clearly decreased by treatment with a PTGER4 receptor agonist, AE1-329, which decrease was almost the same as that seen in PGE_2_-treated cells (Fig. 6A and 6B). Moreover, PGE_2_-mediated suppression of lipid accumulation was cleared by co-treatment with an EP4 receptor antagonist, L-161982, but not with AH6809, a PTGER1 receptor antagonist (Fig. 6A and 6B). Next, we measured the intracellular triglyceride level when the cells were caused to differentiate into adipocytes in medium containing PGE_2_, DI-004 or AE1-329 or PGE_2_ with or without AH6809 or L-161982. Differentiation-mediated enhancement of the intracellular triglyceride level was suppressed by treatment with PGE_2_ or AE1-329, but not with DI-004 (Fig. 6C). Moreover, PGE_2_-mediated decrease in the intracellular triglyceride level was cleared by co-treatment with L-161982, but not AH6809 (Fig. 6C). When 3T3-L1 cells were cultured for 3 h by chemicals, followed by further cultured for 6 days without chemicals, almost the same results as those for 6 days were observed (data not shown).

**Figure 8:**
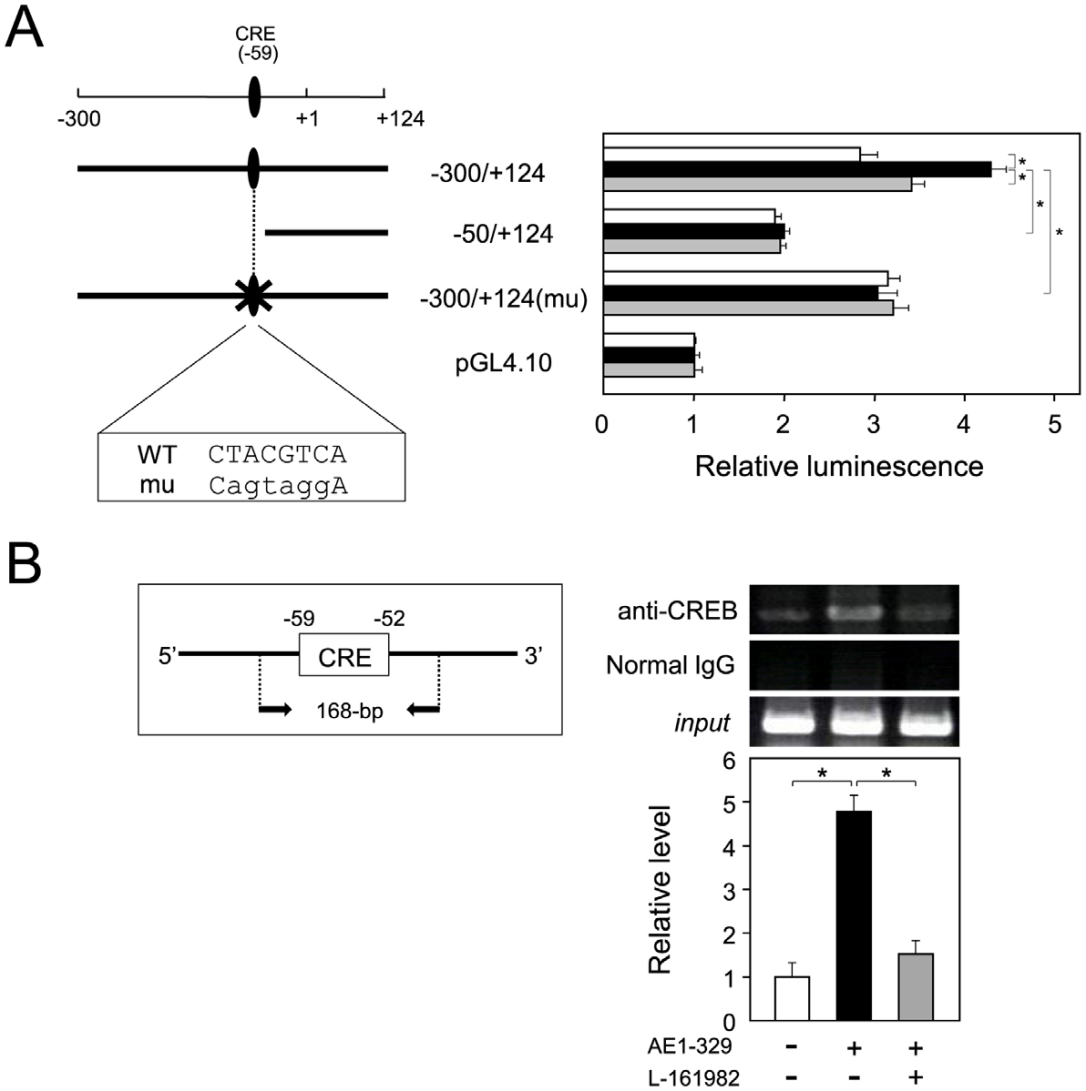
Enhancement of PTGER4 receptor-mediated *Ptgs2* gene expression through binding of CREB to *Ptgs2* promoter. A. Deletion and mutation analyses of the mouse *Ptgs2* promoter region in 3T3-L1 cells. Transfected cells were cultured for 48 h. Luciferase reporter activities were measured (*right panel*) after the cells has been treated with AE1-329 (1 µM, *black column*), AE1-329 and L-161982 (10 µM, *gray columns*) or incubated without any treatment (*white columns*) for 6 h. The data represent the mean ± S.D. of 3 independent assays. The CRE at position −59 is indicated at the *top* of the diagram, and mutated nucleotides are shown by small characters (*left panel*). *p<0.01, as indicated by the brackets. B. ChIP assay of the CRE of the *Ptgs2* promoter in 3T3-L1 cells. The scheme for the ChIP assay for the *Ptgs2* promoter is shown at the *left*. The cells were untreated or treated with AE1-329 (1 µM) with or without L-161982 (10 µM) for 3 h, and the ChIP assay was then carried out. The profile of the amplicon is shown at the *right*. The input control (*input*) means that a small aliquot before immunoprecipitation was used for PCR amplification. The precipitated DNA level was estimated by quantitative PCR analysis as described in the Materials and Methods. The data are representative of 3 independent experiments. *p<0.01, as indicated by the brackets.

Furthermore, when PTGES1 siRNA-transfected cells were caused to differentiate into adipocytes for 6 days with or without DI-004 or AE1-329, Oil Red O staining of the intracellular lipids was carried out and the intracellular triglyceride level was measured. Only AE1-329 could repress the PTGES1 siRNA-mediated enhancement of adipogenesis (Fig. 6D–F). Almost the same results were obtained when the PTGES1 siRNA-transfected cells were treated with PTGER1 or PTGER4 agonist for 3 h, followed by cultured for 6 days in the absence of PTGER1 or PTGER4 agonist (data not shown). These results, taken together, indicate that PTGES1-produced PGE_2_ suppressed adipogenesis by acting through PTGER4 receptors in 3T3-L1 cells.

### Activation of PTGER4 Receptor Enhances PGE_2_ Production with Elevation of *Ptgs2* Expression in 3T3-L1 Cells

When 3T3-L1 cells were cultured with AE1-329, we also found that the production of PGE_2_ was increased approximately 2.8-fold, as compared with that obtained for the vehicle-treated cells (Fig. 7A). The expression of the *Ptgs2* gene was enhanced approximately 4.3-fold at 3 h after the initiation of adipogenesis, as compared with that in the preadipocytes (Fig. 7B). Moreover, when the cells were caused to differentiate into adipocytes in the presence of AE1-329, the expression of the *Ptgs2* gene was elevated about 1.3-fold at 3 h, as compared with that in vehicle-treated cells (Fig. 7B). Then the expression level quickly decreased to a level lower than that detected in preadipocytes (Fig. 7B). Furthermore, the AE1-329-mediated enhancement of the expression of the *Ptgs2* gene was repressed by co-treatment with L-161982 (Fig. 7C, *left panel*). In addition, PGE_2_ itself was able to elevate the expression of the *Ptgs2* gene in 3T3-L1 cells, and this enhancement was suppressed by co-treatment with L-161892 (Fig. 7C, *right panel*). These results indicate that PGE_2_ enhanced its own production by acting through the PTGER4 receptor to elevate the expression of the *Ptgs2* gene in an autocrine manner in 3T3-L1 cells.

**Figure 9:**
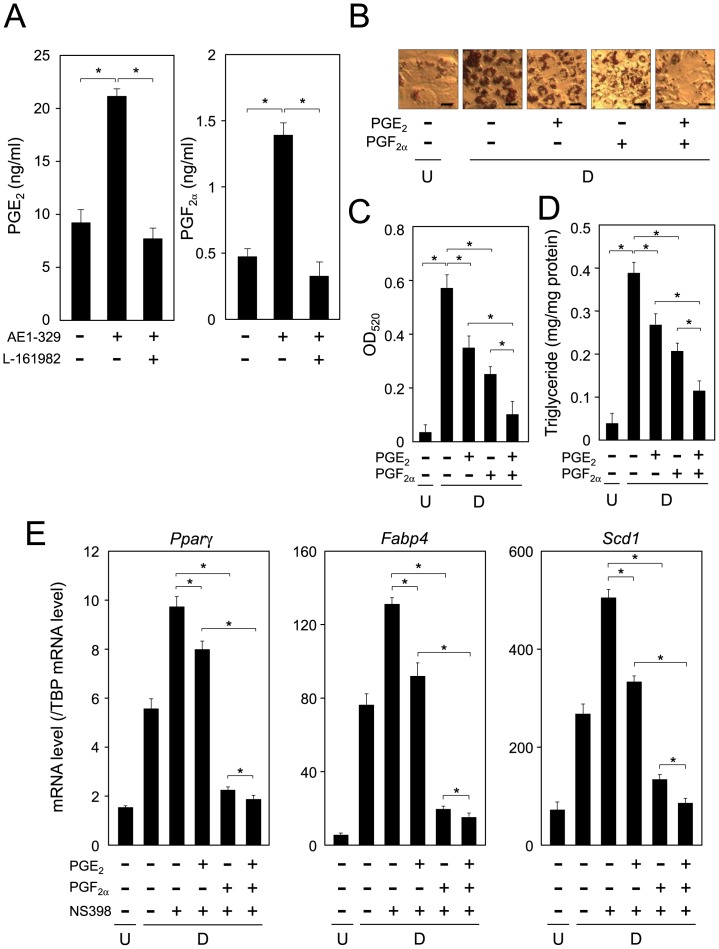
Synergistic suppression of adipogenesis by PGE_2_ and PGF_2α._ A. Enhancement of PGE_2_ and PGF_2α_ production in AE1-329-treated adipocytes. 3T3-L1 cells were incubated for 1 h in DMEM containing or not AE1-329 (1 µM) with or without L-161982 (10 µM). The medium was then removed and replaced with fresh DMEM containing AE1-329 and/or L-161982 and A23187 (5 µM), and the cells were further incubated for 10 min. The medium was collected for the measurement of PGE_2_ and PGF_2α_ levels by performing the respective EIAs. Data are expressed as the mean ± S.D. of 3 independent experiments. *p<0.01, as indicated by the brackets. B. Oil Red O staining. 3T3-L1 cells were caused to differentiate into adipocytes for 6 days in DMEM containing PGE_2_ (100 nM) and/or PGF_2α_ (100 nM; Cayman Chemical). PGE_2_ and PGF_2α_ were added every day. Bar  = 50 µm. C. Measurement of Oil Red O dye extracted from lipid droplet-laden cells. D. Intracellular triglyceride level. 3T3-L1 cells were cultured as described in the legend of Fig. 9B. Data are presented as the mean ± S.D. from 3 independent experiments. *p<0.01, as indicated by the brackets. E. Expression of adipogenic genes. Cells were cultured as described in the legend of Fig. 9B. Transcription levels were measured by quantitative PCR. Data are the mean ± S.D. of 3 independent experiments. *p<0.01, as indicated by the brackets.

### Involvement of CREB in the PGE_2_-mediated Activation of *Ptgs2* Gene Expression

CREB has been identified as the activator for the transcription of the *Ptgs2* gene in 3T3-L1 cells [18]. So, we investigated whether the CREB was involved in the PGE_2_/PTGER4 receptor-elevated *Ptgs2* gene expression by performing a luciferase reporter assay. The transcription initiation site of the mouse *Ptgs2* gene has been determined [28]. When the construct carrying the promoter region from −300 to +124, named −300/+124, was used for the transfection, efficient reporter activity was detected (Fig. 8A). Moreover, when the −300/+124 construct-transfected cells were treated with AE1-329, the luciferase reporter activity was enhanced to become approximately 151% (*black column*) of that of the vehicle (*white column*); and this AE1-329-activated *Ptgs2* promoter activity was suppressed by the co-treatment with L-161982 (*gray column*) to become about 78% of the promoter activity of the AE1-329-treated cells (Fig. 8A). Furthermore, when the region from −300 to −50 was deleted, the luciferase reporter activity was significantly decreased, and the responses to AE1-329 and L-161982 disappeared (Fig. 8A). To confirm the importance of the CRE at position −59 in the PGE_2_-derived elevation of *Ptgs2* gene expression in 3T3-L1 cells, we introduced a mutation at this position in the −300/+124 construct; −300/+124(mu) [18]. When the cells were transfected with this −300/+124(mu) construct, the responsiveness to AE1-329 and L-161982 was lost; although the basal promoter activity was not altered (Fig. 8A). These results indicate that PGE_2_ activated *Ptgs2* gene expression through the CRE at position −59 of the mouse *Ptgs2* promoter in 3T3-L1 cells.

**Figure 10:**
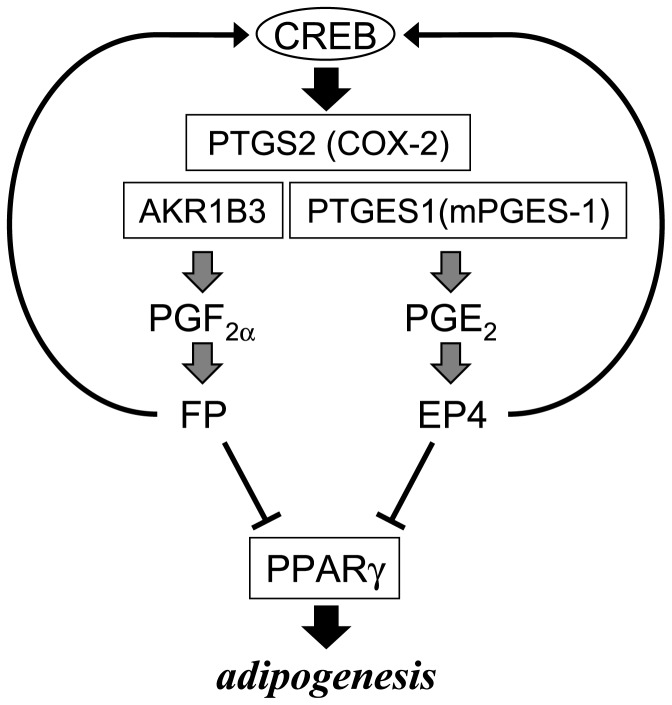
Synergistic suppression of the early phase of adipogenesis by PGE_2_ and PGF_2α_ in 3T3-L1 cells.

Next, we examined the binding of CREB to the CRE at −59 of the *Ptgs2* promoter by performing a chromatin immunoprecipitation (ChIP) assay. The expected size (168-bp; Fig. 8B, *left panel*) of an amplicon containing the CRE at −59 was detected in the formaldehyde-fixed DNA-protein complexes immunoprecipitated with anti-CREB antibody (Fig. 8B, *right panel*). Moreover, when the cells were treated with AE1-329, the binding efficiency was enhanced about 4.7-fold as compared with that of the untreated cells (Fig. 8B, *right panel*), and the AE1-329-derived increase in the efficiency of binding of CREB to the CRE was clearly suppressed by co-treatment with L-161982 (Fig. 8B, *right panel*). On the contrary, there was no detectable signal when rabbit normal IgG was added (Fig. 8B, *right panel*). These results indicate that PGE_2_-mediated upregulation of *Ptgs2* gene expression occurred by enhancing the binding of CREB to the CRE of the *Ptgs2* gene promoter in 3T3-L1 cells.

### Synergistic PGE_2_ and PGF_2α_-mediated Suppression of Adipogenesis

PGF_2α_ and PGE_2_ suppress the progression of adipogenesis through their specific PG receptors, i.e., PTGFR and PTGER4, respectively [18], [20]. Moreover, PGF_2α_ induces the production of anti-adipogenic PGE_2_ and PGF_2α_ by triggering the PTGFR receptor/MEK/ERK cascade in 3T3-L1 cells [18]. PGE_2_ also enhances the production of anti-adipogenic PGE_2_ and PGF_2α_ in mouse embryonic fibroblasts [20].

We examined whether PTGER4 receptor-mediated activation would enhance PGF_2α_ production in 3T3-L1 cells. PGE_2_ production was increased by the treatment with AE1-329, and this enhancement was lost by co-treatment with L-161982, (Fig. 9A). Furthermore, PGF_2α_ production was also enhanced by treatment with AE1-329, and the co-treatment with L-161982 blocked this increase (Fig. 9A). These results reveal that AE1-329-derived activation of the PTGER4 receptor enhanced *de novo* synthesis of anti-adipogenic PGE_2_ and PGF_2α_ in 3T3-L1 cells.

As both PGE_2_ and PGF_2α_ act as anti-adipogenic PGs in adipocytes, we investigated their suppression effects on adipogenesis in 3T3-L1 cells. When the cells were caused to differentiate into adipocytes for 6 days in medium containing either PGF_2α_ or PGE_2_ along with NS-398, which is an inhibitor of PTGS2 and thus suppresses the *de novo* synthesis of PGs including the anti-adipogenic PGE_2_ or PGF_2α_, the accumulation of the intracellular lipids shown by Oil Red O staining was decreased as compared with that in the vehicle-treated cells (Fig. 9B and 9C). In addition, greater suppression was observed when the cells were cultured in medium containing both PGF_2α_ and PGE_2_ (Fig. 9B and 9C). The intracellular triglyceride level was elevated during adipogenesis, and the enhanced triglyceride level was suppressed by co-treatment with either of PGE_2_ and PGF_2α_. Moreover, when the cells were caused to differentiate into adipocytes in medium containing both PGE_2_ and PGF_2α_, the intracellular triglyceride level was lower than that in PGE_2_ or PGF_2α_-treated cells (Fig. 9D).

Next, we measured the expression level of adipogenic genes in PGE_2_- and/or PGF_2α_-treated cells. When 3T3-L1 cells were caused to differentiate into adipocytes, the expression levels of *Pparγ*, *Fabp4*, and *Scd1* genes were enhanced approximately 3.1-, 7.1-, and 3.3-fold, respectively, as compared with those in the undifferentiated cells (Fig. 9E). In addition, the expression levels of these genes were enhanced even more in adipocytes cultured in medium containing NS-398. When the cells were caused to differentiate into adipocytes in medium containing both PGE_2_ and PGF_2α_, the expression levels of the genes were decreased to a greater extent than when the cells were cultured in medium containing PGE_2_ or PGF_2α_ (Fig. 9E). Furthermore, the suppression effect on adipogenesis by PGE_2_ was weaker than that by PGF_2α_ (Fig. 9E). These results indicate that PGE_2_ or PGF_2α_ synergistically suppressed adipogenesis in 3T3-L1 cells.

## Discussion

PGs are known to be involved in the regulation of adipogenesis. PGD_2_ is synthesized by lipocalin-type PGD synthase in adipocytes and accelerates the mid-late phase of adipogenesis [11]. PGI_2_ is involved in the activation of preadipocytes to adipocytes through PGI_2_ receptor [14], [15]. In contrast, PGF_2α_ and PGE_2_ suppress the progression of adipogenesis [17], [18], [19], [20]. PGF_2α_ is synthesized by AKR1B3 in adipocytes and represses the early phase of adipogenesis by engaging the PTGFR receptor [17]. Moreover, PGF_2α_ enhances the production of itself and PGE_2_ by enhancing the expression of the COX-2 gene via activation of the PTGFR receptor-ERK/CREB cascade [18]. PGE_2_ also acts as anti-adipogenic factor, by acting through the PTGER4 receptor [19]. A recent study demonstrated that PGE_2_-PTGER4 signaling suppresses adipocyte differentiation by negatively affecting *Pparγ* expression in an autocrine manner in adipocytes [20]. However, the PGE_2_-producing enzyme in adipocytes and the precise mechanism regulating the suppression of adipogenesis by PGE_2_ have not been fully understood. Here, we found that PTGES1 synthesized PGE_2_ in 3T3-L1 cells, which PG then suppressed the early phase of adipogenesis via the PTGER4 receptor. Moreover, PGE_2_ enhanced *Ptgs2* gene expression through the positive feedback loop via PTGER4 receptor, and the elevated PGE_2_ and PGF_2α_ production. Furthermore, AKR1B3-produced PGF_2α_ suppresses the early phase of adipogenesis through PTGFR receptor [17], and increased the expression of the *Ptgs2* gene [18], like PGE_2_. Thus, PTGES1-produced PGE_2_ and AKR1B3-synthesized PGF_2α_ synergistically suppress the progression of the early phase of adipogenesis (Fig. 10).

Until now, three major enzymes that catalyze the production of PGE_2_ from PGH_2_ have been identified [21], [29]: PTGES1 [23], PTGES2 [25], and PTGES3 [26]. PTGES1 has been identified as the member of the MAPEG family [30]. These three PTGESs were constitutively expressed during adipocyte differentiation of 3T3-L1 cells (Fig. 2). When the expression of PTGES1 was suppressed by its siRNA, the PGE_2_ level was significantly decreased (Fig. 3A and 3B), indicating that PTGES1 was the PGES active in adipocytes. There are two different papers in the literature concerning the expression of PTGES1 in adipocytes. Hetu *et al.* reported that PTGES1 levels in obese fats are significantly lower than those in lean animals [31]. However, the other report by Xie *et al.* indicated that PTGES1 is enhanced during differentiation of 3T3-L1 cells [32], thus differing from our results. At the present time, there is no clear explanation for this discrepancy. Further precise studies of the *in vitro* and *in vivo* functions of PTGES1 in adipocytes are needed to solve this problem. In addition, we have to also elucidate the effects of GST activity of PTGES1 in the regulation of adipogenesis, because PTGES1 also carries GST activity [33].

PG synthesis is coordinately regulated through the coupling of terminal PG synthases with each or both of PTGS1 (COX-1) and PTGS2 [34]. Both PTGSs were expressed in the undifferentiated 3T3-L1 cells (data not shown), indicating that both PTGSs would probably have the ability to couple with PTGES1 for the production of PGE_2_. PTGES1 is co-localized with both PTGS isozymes in the perinuclear envelope [35]. However, PTGES1 is functionally coupled with PTGS2 to produce PGE_2_ [35]. In fact, the PGE_2_ production profile well resembled the expression profile of the *Ptgs2* gene (Fig. 2). PGs are known to be associated with *Ptgs2* gene expression in an autocrine manner in a variety of cells including adipocytes [18], [20], [36], [37]. PGF_2α_ increases the expression of the *Ptgs2* gene via the PTGFR/ERK/CREB cascade, which increase is followed by elevation of PGF_2α_ and PGE_2_ production in 3T3-L1 cells [18]. PGE_2_ also enhances the production of PGE_2_ and PGF_2α_ through the PTGER4 receptor in mouse embryonic fibroblasts [20] and suppresses the progression of adipogenesis [19]. Anti-adipogenic PGF_2α_ and PGE_2_ increased themselves to enhance the suppression of adipogenesis in the early phase of adipogenesis. However, the suppression of adipogenesis by these anti-adipogenic PGs was terminated within several hours after the initiation of adipogenesis. Therefore, the molecular mechanism underlying this termination needs be further elucidated.

In summary, we identified PTGES1 (mPGES-1) as the PGES active in adipocytes, whose expression was detected in both preadipocytes and adipocytes during adipogenesis. PTGES1-synthesized PGE_2_ suppressed the early phase of adipocyte differentiation via the PTGER4 (EP4) receptors by down-regulating adipogenic gene expression. Furthermore, PGE_2_ enhanced the expression of the *Ptgs2* (*COX-2*), and induced the production of itself and PGF_2α_. Both anti-adipogenic PGs synergistically suppressed the progression of adipogenesis in 3T3-L1 cells. Further studies will be needed to elucidate the *in vivo* functions of PGE_2_ and PGF_2α_ in the suppression of obesity.
